# Understanding and Overcoming the Fundamental Chemical and Electronic Challenges of SnBr_4_ Impurities in Tin Perovskite Solar Cells

**DOI:** 10.1002/smsc.202500426

**Published:** 2025-11-11

**Authors:** Amanz Azaden, Thomas Webb, Polina Jacoutot, Harry Spear, Robert Palgrave, Saif A. Haque

**Affiliations:** ^1^ Department of Chemistry and Centre for Processable Electronics Molecular Sciences Research Hub Imperial College London White City London W12 0BZ UK; ^2^ Department of Chemistry University College London 20 Gordon St London WC1H 0AJ UK

**Keywords:** defects, photochemistry, solar cells, tin perovskites

## Abstract

Tin perovskite solar cells (Sn‐PSCs) have emerged as excellent candidates for nontoxic narrow bandgap PSCs. Nevertheless, the technology remains limited by both stability and suboptimal energetic alignment with conventional charge transport layers. Compositional tuning is central to high‐performance Sn‐PSCs, replacing substoichiometric iodide ions with bromide. However, incorporating SnBr_2_ as the bromide source introduces SnBr_4_ impurities, underscoring the need to understand the consequences of SnBr_4_ on both performance and degradation chemistry. Presently, the absence of such understanding has engendered a reliance on organobromide salts, neglecting a critical opportunity to enhance stability via the reduction of unstable SnI_2_. Herein, the influence of SnBr_4_ impurities on the structural, optoelectronic, and electronic properties of Sn‐perovskites is investigated. Removal of SnBr_4_ impurities from SnBr_2_ results in drastically improved morphology, a 40% lower trap density and enhanced device performance of 150%. Furthermore, both the fundamental chemistry and degradation pathways in SnI_4_ and SnBr_4_ are compared, demonstrating the latter does not decompose to the molecular halogen—a key weakness of iodine‐based Sn‐PSCs. The present findings offer critical chemical and electronic insights into the presence of SnBr_4_, the importance of its removal and the opportunities afforded by using SnBr_2_ to minimize unstable SnI_2_ in Br‐rich Sn‐perovskite phases.

## Graphical abstract

Halide substitution has emerged as an essential channel to realize high‐performance tin perovskite solar cells, achieved largely using organohalides. Herein, the inorganic counterpart SnBr_2_ is investigated, revealing the detrimental impact of associated SnBr_4_ states, which can be isolated using a simple purification. Such removal engenders enhanced optoelectronic properties and device performance, emphasizing the critical impact of SnBr_4_.

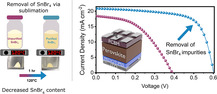

## 1 Introduction

Inorganic‐organic perovskite solar cells (PSCs) have garnered significant interest in the last decade due to their remarkable rise in power conversion efficiencies (PCEs) over a short space of time (3.8% to 27%).^[^ ^1^, ^2^, ^3^ ^]^ Such advances are attributed to their ideal optoelectronic properties, namely strong optical absorption, high carrier mobilities, and compatibility with low‐cost, large‐area fabrication techniques.^[^ ^4^, ^5^, ^6^ ^]^ However, to date, the highest‐performing PSCs utilize lead (Pb) cations, raising concerns of toxicity which in turn impedes the commercialization of these materials.^[^ ^7^, ^8^, ^9^, ^10^ ^]^ To address this issue, research activity into alternative absorbers to Pb‐based perovskites has identified Sn‐based perovskites as excellent candidates to mitigate toxicity whilst boasting bandgaps closer to the ideal bandgap of a narrow bandgap absorber (1.3–1.4 eV).^[^ ^11^ ^]^

Nevertheless, Sn‐based perovskites remain hindered by the well‐documented instability of Sn(II) and its facile oxidation to an Sn(IV) state.^[^ ^11^, ^12^, ^13^ ^]^ Previously we have shown that in SnI_2_‐based perovskites this oxidation promotes a self‐sustained degradation cycle where iodine (I_2_), once evolved, catalyzes oxidation thereby accelerating degradation.^[^ ^9^ ^]^ Considering the unstable Sn(II), strategies to improve resistance to oxidation have included solvent engineering, additive engineering, and purification of the SnI_2_ precursor salt.^[^ ^9^, ^14^, ^15^, ^16^, ^17^, ^18^, ^19^, ^20^, ^21^, ^22^ ^]^ Specifically, regarding purification, improvements have been realized via thermal sublimation in inert environments, recrystallization of SnI_2_, toluene‐washing, and the formation of DMSO:SnI_2_ adducts in situ.^[^ ^9^, ^17^, ^23^, ^24^, ^25^ ^]^ These approaches have been shown to reduce SnI_4_ concentrations in the precursor and enhance the device performance of Sn‐PSCs.^[^ ^9^, ^17^, ^23^, ^24^, ^25^ ^]^ Using this approach, we previously demonstrated the direct impact of SnI_4_ within the perovskite and outlined that high‐purity SnI_2_ is essential in the fabrication of high‐performance solar cells.^[^ ^9^ ^]^ This has in turn prompted a range of studies targeting SnI_4_ to better understand and manage the critical role of SnI_4_ on both stability and performance.^[^ ^9^, ^17^, ^23^ ^]^ We therefore suggest that an alternative means to reduce this cyclic degradation is to reduce the fraction of SnI_2_ in the starting precursor, coined “halide substitution”.

To date, the rationale for Br incorporation in Sn‐PSCs is to address the energy level mismatch between Sn‐based PSCs and conventional charge transport layers, which exists due to the shallower valence band in Sn‐perovskites. Modification of the valence band of the Sn‐perovskite by compositional substitution on the halide X‐site can address this misalignment by deepening the valence band, improving both charge injection and photovoltage.^[^ ^26^, ^27^, ^28^, ^29^, ^30^, ^31^ ^]^ Halide substitution in Sn‐perovskites may be achieved via the inclusion of either organobromide salts (MABr, FABr, GABr, PEABr), else via the metal halide, SnBr_2_. In contrast to SnI_2_ chemistry and its subsequent decomposition into SnI_4_ and iodine, there has been very little reported on the chemistry of SnBr_2_ and the implications of SnBr_4_ on Sn‐perovskites. This gap in understanding within the literature can be partly attributed to a dependency on the use of organobromide salts as a preferred choice for Br inclusion in Sn‐perovskites, especially for inclusion at substoichiometric quantities.^[^ ^28^, ^30^, ^32^, ^33^ ^]^ Interestingly, we note the discrepancy between Sn and Pb‐based perovskites, whereby PbBr_2_ is frequently incorporated into the precursor to produce some of the highest‐performing devices (including champion p‐i‐n performances),^[^ ^3^, ^34^, ^35^, ^36^ ^]^ demonstrating a clear difference in fabrication strategy between the two systems. This contrast in approaches between the use of the metal‐bromide salts in Pb and Sn‐perovskites most likely originates from the greater understanding of the importance of a high‐purity SnI_2_ precursor, most closely following the existing narrative on iodine‐based degradation chemistry.^[^ ^26^, ^27^, ^28^, ^29^, ^30^ ^]^ This engenders two important challenges that cannot be addressed via the use of organobromides, i) the fabrication of perovskites with a high Br content exceeding a third by stoichiometry, and ii) the opportunities to substitute out problematic SnI_2_, which undergoes facile oxidation and releases I_2_. Considering the former, larger fractions of Br have useful application as wide bandgap absorbers, recently applied in tandem photovoltaics,^[^ ^37^ ^]^ as well as LEDs and other optoelectronic applications.^[^ ^32^, ^33^ ^]^ We note that both of these challenges can be addressed through a better understanding of the prevalence of SnBr_4_, and its effect on the material, optical, and electronic properties, as well as perhaps most crucially, the implications on the intrinsic chemical stability. Indeed, understanding these factors in the SnI_4_ has proved crucial in encouraging better design and performance in pure iodide Sn‐PSCs, consolidating the need for an analogous study with SnBr_2_.

In this work, we first demonstrate a simple, one‐step purification technique of subliming the precursor, which lowers the concentration of SnBr_4_ within the precursor by a factor of 10. This reduction in SnBr_4_ provides the opportunity to probe the effect of trace SnBr_4_ species on key material, optical, and photovoltaic parameters. To this end, we observe a significant increase in carrier lifetime, emission intensity, and PCE enhancements from 3.3% to 8.5% when ensuring SnBr_4_ is minimized from the precursor. In doing so, we present our findings on the feasibility of SnBr_2_, via the isolation and removal of SnBr_4_ impurities. To demonstrate these findings, we prepare perovskites with a Br:I stoichiometry exceeding the maxima of 1:2 achievable through the sole use of organobromides.^[^ ^17^, ^32^, ^38^, ^39^, ^40^, ^41^, ^42^ ^]^ Significantly, having established that removal of SnBr_4_ is essential to the device performance, we next demonstrate improved ambient stability of SnBr_2_ in comparison to SnI_2_, justifying its substitution with SnI_2_ tin precursor salt. In a key result, we find that whilst SnI_2_ undergoes a chemical decomposition to I_2_, a potent and highly destructive degradation product, SnBr_2_ does not undergo an analogous decomposition to form Br_2_. Hence, this makes the application of SnBr_2_ as a bromide ion source attractive not only for valence band alignment, but crucially for reducing the fraction of unstable SnI_2_, thereby proportionally limiting I_2_‐related degradation pathways, an advantage that organohalides cannot offer.

## 2 Results and Discussion

### 2.1 Purification of SnBr_2_

**Figure 1:**
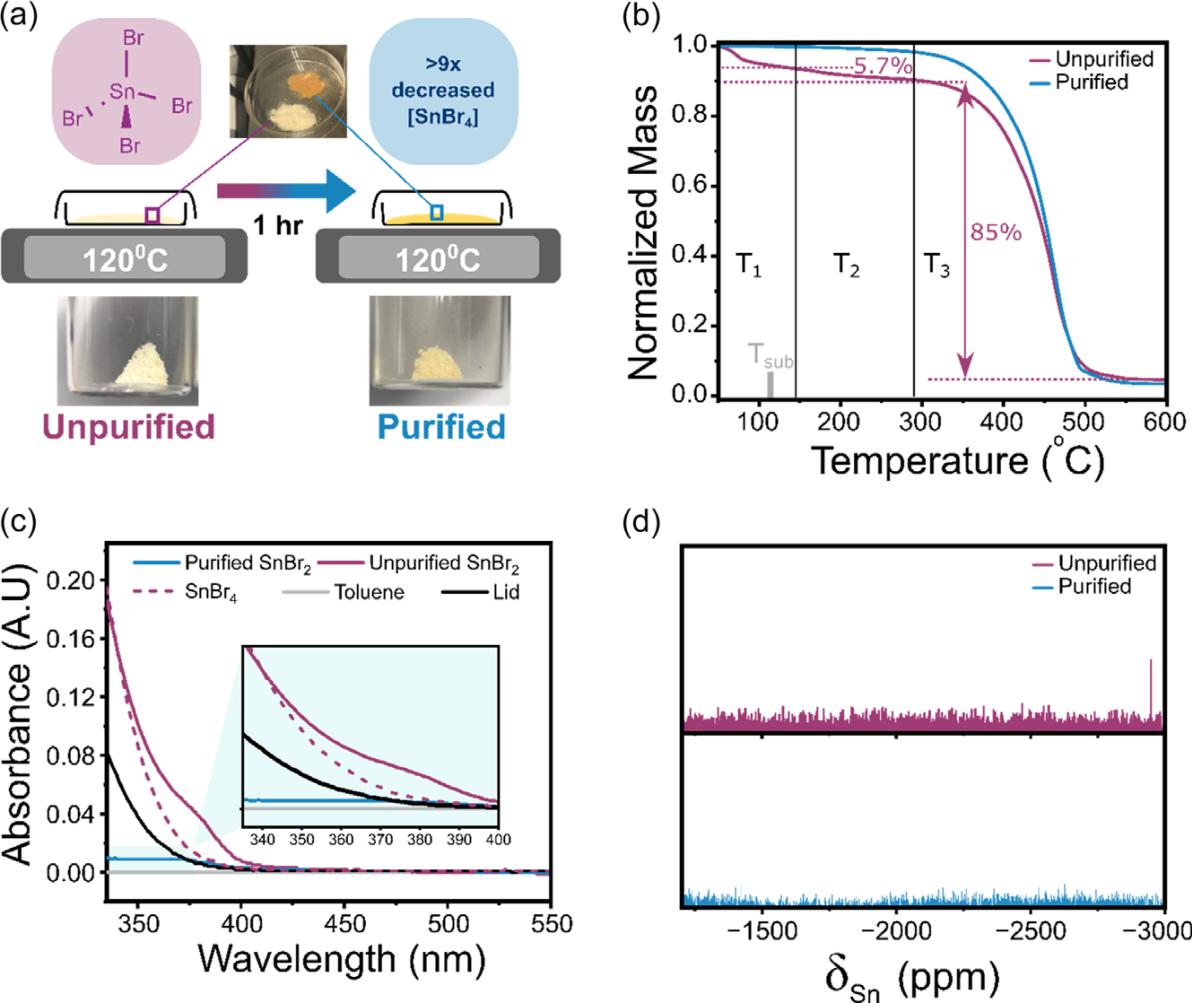
Chemical analysis of thermal treatment on SnBr_2_. a) Schematic showing the sublimation process; SnBr_2_ is heated in an inert environment for 1 hr at 120 °C, showing a color change from pale yellow to orange. b) TGA of purified and unpurified SnBr_2_ powders. c) UV–visible absorbance spectrum of a solution of purified and unpurified SnBr_2_ powders dissolved in toluene, in addition to a control SnBr_4_ and toluene. d) ^119^Sn NMR spectra of toluene used to wash SnBr_2_ before and after purification. Peak in unpurified sample corresponds to removed SnBr_4_; no SnBr_4_ is observed after purification, sample diluted with 20% v/v CDCl_3_ to provide a lock signal.

We begin by investigating the potential of using thermal sublimation treatments to separate trace SnBr_4_ from commercial SnBr_2_ precursor materials. To do this, commercial SnBr_2_ powders were sublimed in an N_2_ glovebox (O_2_ < 1 ppm) for 1 h at 120 °C (T_Sub_) shown in **Figure** **1**a,b. This procedure resulted in an obvious color change in the SnBr_2_ powder from pale yellow to pale orange. This change was accompanied by the formation of a film on the lid of the Petri dish. From herein, we will refer to the as‐purchased SnBr_2_ as “unpurified”, whilst the powder treated at 120 °C for 1 hr is referred to as “purified”.

As seen in Figure 1b, thermogravimetric analysis (TGA) of both SnBr_2_ powders shows three distinct phases. In the first lower temperature phase (T_1_ < 140 °C), we observe a decrease in mass within the unpurified SnBr_2_ of 5.7%, significantly larger than the 0.1% recorded in the purified equivalent. This initial mass loss can be attributed to the removal of SnBr_4_ (confirmed using TGA, Figure S1a, Supporting Information, showing two mass loss phases attributed to SnBr_4_ and H_2_O given its hygroscopic nature) and is consistent with the lower enthalpy of sublimation of SnBr_4_ (63.8 kJ mol^−1^) when compared to SnBr_2_ (134.6 kJ mol^−1^), as well as vapor pressures reported in the literature,^[^ ^43^ ^]^ and thus a lower sublimation temperature.^[^ ^44^, ^45^ ^]^ Increasing the temperature further, the rate of mass loss slows, and a plateau is observed, indicating that impurities with low sublimation temperatures have been removed. Using this data, we propose this region provides a useful range of temperatures for the purification of SnBr_2_ (140 °C < T_2_ < 300 °C). At temperatures exceeding 300 °C the samples both undergo a second mass loss, attributed to the sublimation of SnBr_2_.

To identify the impurity removed during purification, UV–visible absorbance spectroscopy was used to both identify and quantify the concentration of the suspected SnBr_4_ impurity. Toluene selectively dissolves Sn(IV) halides and has been used extensively in studies surrounding SnI_4_ degradation work.^[^ ^9^, ^13^ ^]^ For this investigation, 3 mL of toluene was added to 3.2 mg of SnBr_2_ powders before and after purification. Figure 1c shows the onset of an absorption feature at ≈380 nm in the unpurified SnBr_2_. Comparison of the absorbance feature with a reference solution of SnBr_4_ confirms the presence of an initial SnBr_4_ component within the unpurified SnBr_2_. Crucially, the impurity is confirmed to be removed following purification, explicated by the now‐absent absorbance feature corresponding to SnBr_4_. This finding is consistent with the absence of mass loss in the T_1_ region of the TGA in samples following purification, and the visible color change of the powder following the treatment, with the latter likely attributed to the removal of the white‐colored SnBr_4_. Moreover, analysis of the impurity material collected on the lid during sublimation matches well with the absorbance profile of SnBr_4_, directly confirming its removal. To obtain further evidence for the loss SnBr_4_, ^119^Sn nuclear magnetic resonance (NMR) spectra were collected using toluene to extract SnBr_4_ before and after purification, relying on the same solvent orthogonality of the impurity species (Figure 1d). Using this principle we compare the Sn‐NMR spectra and observe the removal of a single Sn species with a chemical shift of ≈−2950 ppm, further identifying SnBr_4_ as the species removed. Having identified SnBr_4_ as an impurity present within SnBr_2_ precursors, we next return to the absorbance spectra to provide a more quantitative estimate of the abundance. To do this, the percentage of SnBr_4_ within the starting material was quantified using the Beer–Lambert (Figure S1b,c, Supporting Information). From serial dilutions of SnBr_4_ in toluene, we report a molar extinction coefficient (*ε*) of SnBr_4_ of 1416 M^−1^ cm^−1^ at a wavelength of 335 nm. Using this value, we calculate masses of SnBr_4_ of 0.18 mg (5.6%) and 0.02 (0.6%) mg for unpurified and purified SnBr_2_, respectively. The values attained are a close match to that of the TGA analysis (5.6% vs. 5.7%), confirming the applicability of these techniques to quantify the Sn(IV) content, and further emphasize the importance of understanding of the composition of SnBr_2_ when used to fabricate Br‐containing high‐quality Sn‐perovskites. Using this analysis, we estimate an ≈5.6 mol% concentration of SnBr_4_ in the unpurified precursor, which provides the basis of further comparison in the following sections of the work.

### 2.2 The Effect of SnBr_4_ Removal on Structural Properties of PEA_0.2_FA_0.8_Sn(I_0.9_Br_0.1_)_3_ Films

**Figure 2:**
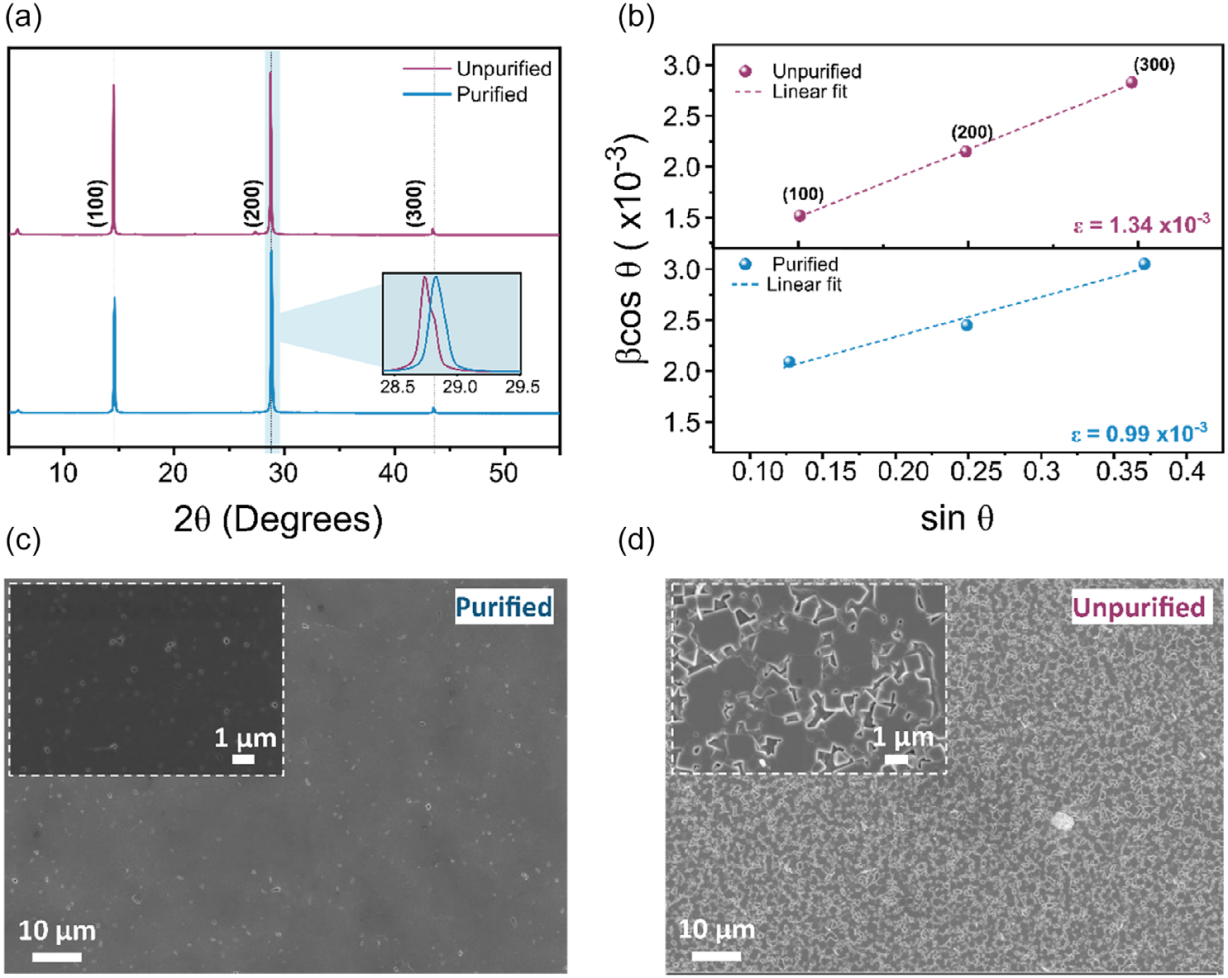
Morphological information of PEA_0.2_FA_0.8_Sn(I_0.9_Br_0.1_)_3_ prepared with purified and unpurified SnBr_2_ deposited on glass. a) XRD diffraction pattern, with major peaks labeled in bold. Inset shows zoomed (200) peak. b) Derived Williamson–Hall plots from the (h00) planes, with a linear fit (dashed line) used to derive strain (bottom right of the plots). c,d) SEM of top view films at 1k × magnification and 10k × magnification (inset).

We begin by investigating the impact of the identified SnBr_4_ impurities, and their removal, on both crystallographic and morphological properties of the resultant perovskite films. To do this, PEA_0.2_FA_0.8_Sn(I_0.9_Br_0.1_)_3_ films were fabricated using typical solution processing methods (see experimental Supporting Information). The impact of purification on the lattice crystallinity was first investigated using X‐ray diffraction (XRD) techniques, revealing an orthorhombic crystal structure of the *Amm*2 space group both with and without purification of the SnBr_2_ salt, which is consistent with literature employing SnBr_2_ as the bromide source for halide substitution.^[^ ^32^, ^33^, ^46^ ^]^ Comparing the diffraction pattern between the perovskite films prepared with and without purification of SnBr_2_, we observe a minor reduction in the crystal lattice unit following the removal of SnBr_4_ (**Figure** **2**a, inset), a shift also seen for the (100) and (300). A reduction in the crystal lattice size following the purification can be attributed to the removal of tensile strain needed to accommodate Sn^4+^ sites within the lattice. These sites have been assigned in the literature to the initial Sn^4+^ ions which are known to form double‐perovskite FA_2_SnX_6_ phases.^[^ ^9^, ^47^, ^48^ ^]^ To investigate the effect of SnBr_4_ impurities on the perovskite microstrain (*ε*_micro_) within the system, Williamson–Hall plots were constructed using the characteristic (100), (200), and (300) diffraction peaks. The lattice strain was found to reduce from *ε*_micro_ = 1.34 × 10^−3^ to *ε*_micro_ = 0.99 × 10^−3^. Both the decreased size of the unit cell and the lower computed lattice strain are indicative of a higher quality, lower defect crystal lattice in films prepared via the purification of SnBr_2_. Specifically in the case of Sn‐perovskites, these defects can be attributed to a combination of vacancy‐ordered double‐perovskite phases and Sn^2+^ Schottky vacancies, which are introduced into the perovskite as charge compensation for excess Sn^4+^ states.^[^ ^9^, ^47^, ^48^, ^49^ ^]^ As such, the presence of even low quantities of SnBr_4_ can have pronounced implications on the crystallographic order and defect chemistry of the perovskite structure.

Scanning electron microscopy (SEM) images were collected to investigate the surface morphology of the films prepared using purified and unpurified SnBr_2_. From the images presented in Figure 2c,d, we observe a significant disruption in the surface morphology in samples containing SnBr_4_ impurities, with purified samples exhibiting significantly reduced pinholes and less pronounced grain boundaries, commensurate with the cross‐sectional images (Figure S16, Supporting Information). In contrast, the unpurified sample is shown to be pinhole‐rich with a greater number of grain boundaries, which are known to inhibit charge carrier transport, facilitate nonradiative recombination and provide a low‐energy surface for degradation to occur.^[^ ^9^ ^]^ Consistent with the crystallographic data, these findings are attributed to the inability of Sn(IV) states to occupy Sn(II) lattice sites, engendering an increased strain and morphological defects. As such, this demonstrates that the removal of SnBr_4_ is essential for high‐quality film morphology when integrating SnBr_2_ as an alternative precursor bromide source to organobromide salts. From these findings, we expect the identified crystallographic and morphological disorder generated by low quantities of SnBr_4_ (5.6 mol%) to have a significant effect on the optoelectronic properties and ultimately photovoltaic performance.

### 2.3 The Effect of SnBr_4_ Removal on Optoelectronic Properties of PEA_0.2_FA_0.8_Sn(I_0.9_Br_0.1_)_3_ Films

**Figure 3:**
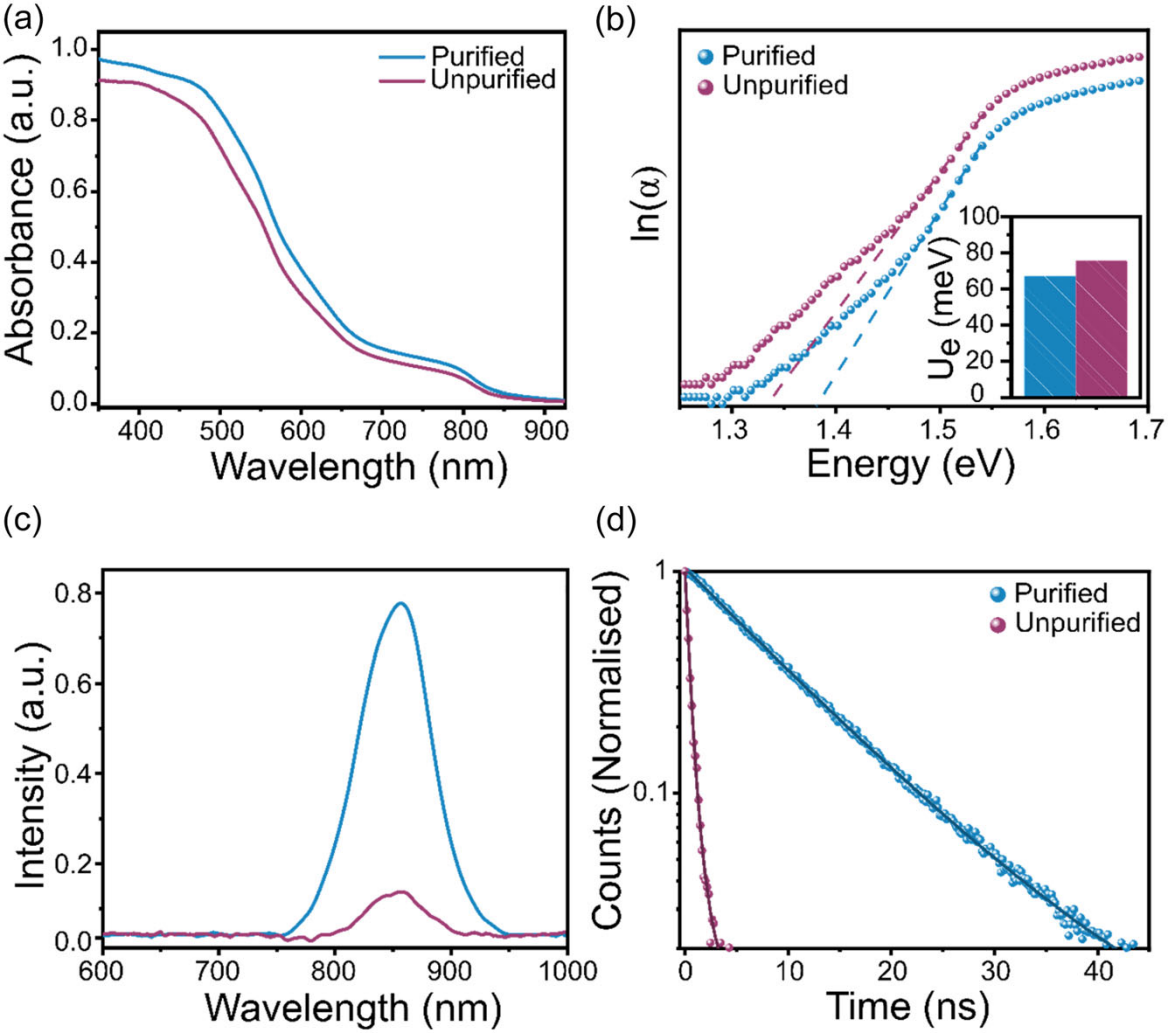
Optoelectronic properties of PEA_0.2_FA_0.8_Sn(I_0.9_Br_0.1_)_3_ prepared with purified and unpurified SnBr_2_ deposited on glass. a) UV–visible absorbance spectrum. b) Urbach plot, where gradients (dashed line) are used to calculate the Urbach Energy (inset). c) Steady state PL spectrum with 404 nm excitation. d) TCSPC, following 404 nm excitation.

We next consider the effect of removing SnBr_4_ from the SnBr_2_ starting material on the optoelectronic properties of perovskite films. UV–visible absorbance (**Figure** **3**a) spectroscopy shows a slight increase in absorbance in films prepared using purified SnBr_2_ and a sharper absorption onset. A sharper absorbance onset has been previously attributed to a lower density of defects at energy states near the band edge.^[^ ^50^ ^]^ Estimation of the Urbach energy (Figure 3b), derived from the absorption onset to infer the presence of defects, revealed a small decrease from 76 to 68 meV following purification of the SnBr_2_, indicative of reduced electronic disorder from fewer Sn^4+^ states, and has been shown to correlate inversely to carrier mobility and voltage deficits in PSCs.^[^ ^33^, ^51^ ^]^ Tauc plots of the absorbance spectra (Figure S2, Supporting Information) also suggest a slight widening of the bandgap (+13 meV) in the unpurified sample. We attribute this to the Moss–Burnstein effect, where the introduction of Sn(IV) states implements p‐type character, consistent with the known self‐doping behavior of Sn‐PSCs.^[^ ^9^, ^12^, ^46^ ^]^

Photoluminescence (PL) spectra shown in Figure 3c exhibit a single emission peak at 860 nm, consistent with emission spectra of similar compositions prepared using organobromide salts as a bromide source, with no apparent peak shift consistent with the near identical bandgap observed in Figure S2, Supporting Information.^[^ ^28^, ^30^ ^]^ Comparing the PL intensity of the perovskites with and without SnBr_4_ removal, we observe a 7.5× increase in emission intensity, indicating a severe adverse effect of SnBr_4_, even at low concentrations. These findings are consistent with our discussion and highlight that the introduction of defects identified promotes nonradiative recombination of carriers, a key loss mechanism in Sn‐PSCs.^[^ ^9^, ^18^, ^23^ ^]^ The correlation between SnBr_4_ and nonradiative recombination was further confirmed using time‐correlated single‐photon counting (TCSPC) measurements (Figure 3d). Fitting the decay of the perovskite excited state yields a significant improvement in the average lifetime from 0.61 to 10.16 ns (Table S1, Supporting Information) following removal of SnBr_4_ impurities. To explicitly establish SnBr_4_ as the direct cause of increased nonradiative recombination pathways, an additional sample was prepared where 5% of SnBr_4_ was deliberately introduced to the purified SnBr_2_ precursor. Consistent with our findings, the films prepared with an additional 5% SnBr_4_ (Table S1 and Figure S3, Supporting Information) exhibited a significantly shorter lifetime of 0.50 ns from TCSPC, which we attribute to the reintroduction and high density of Sn^4+^ trap states.

**Figure 4:**
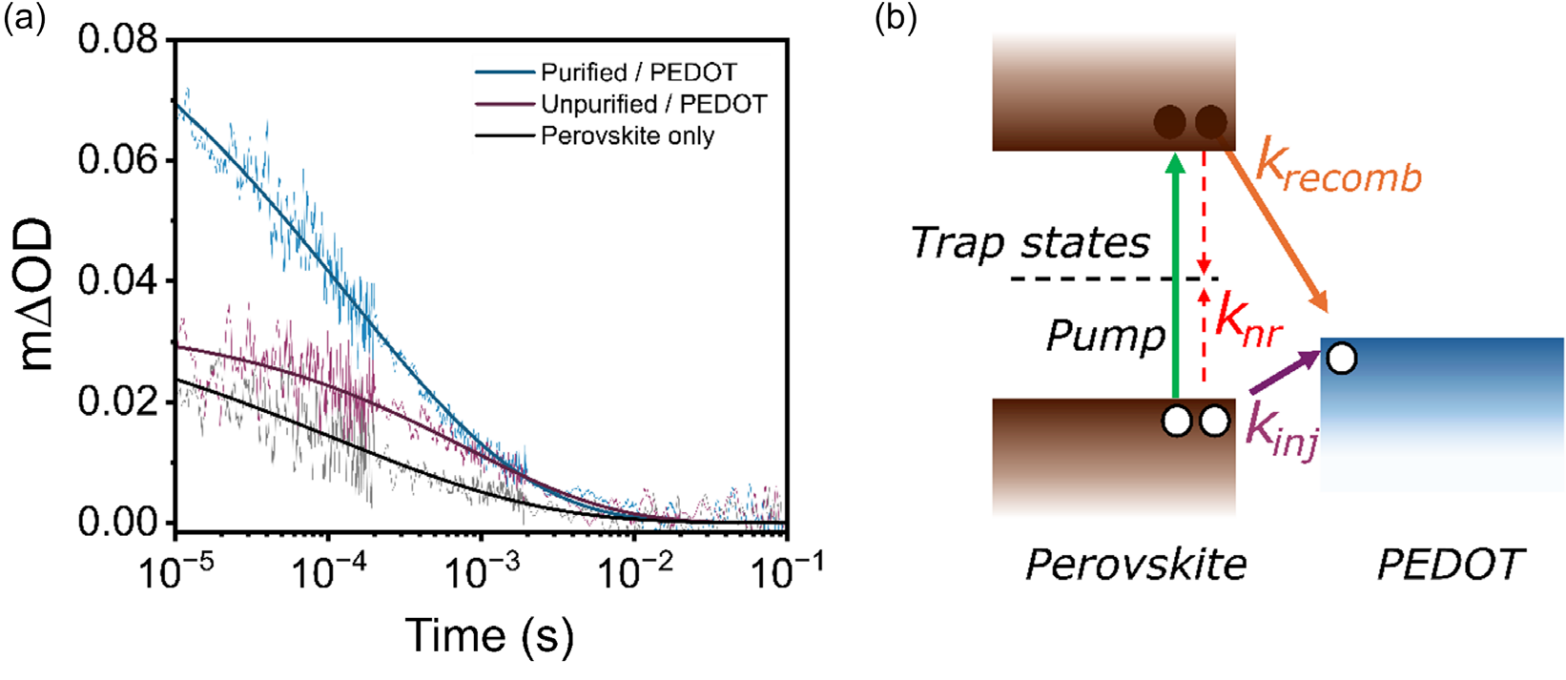
TAS analysis of the effect of purification on hole extraction. a) μs‐TAS analysis of perovskite/PEDOT interface. Fitted transient absorption decay of hole extraction into PEDOT with and without purification of the perovskite and b) schematic of carrier recombination mechanisms.

To further corroborate the effect of Sn^4+^ traps on the kinetics of nonradiative recombination and charge extraction into ahole transport layer (HTL), transient absorption spectroscopy (TAS) was carried out (**Figure** **4**a). TAS measurements offer useful information regarding the yield of hole injection (*k*_inj_) and subsequent back‐charge recombination with electrons from the perovskite conduction band (*k*_recomb_), provided a change in optical density (Δ*OD*) occurs within the HTL following hole injection (Figure 4b).^[^ ^52^, ^53^, ^54^, ^55^, ^56^, ^57^, ^58^ ^]^ Comparing the wavelength dependence of Δ*OD* in PEDOT with changes in the absorbance spectra following chemical oxidation, we find 1600 nm is a suitable wavelength for probing the bipolaron of PEDOT (Figure S4, Supporting Information). Using this approach, we prepare perovskite films with and without purification of SnBr_2_ onto a mesoporous TiO_2_ film (electron acceptor) before depositing a layer of PEDOT on top of the perovskite absorber. From the decays we observe a significant increase in the maximum change in optical density (Δ*OD*_max_) following purification, indicating a higher yield of holes extracted into the HTL.^[^ ^54^, ^56^, ^59^, ^60^ ^]^ This is consistent with the smaller nonradiative recombination rate constant (*k*_nr_) value observed within the PL and time resolved photoluminescence allowing for a greater yield of hole injection as observed within the TAS measurements.

We next estimate the recombination lifetime (*τ*_recomb_ = 1/*k*_recomb_) by determining the time taken for the transient signal to decay to 50% of its original value at 10 μs, which we define as the Δ*OD*_max_. Comparing the decay lifetimes (*τ*_recomb_), we observe a significantly shorter lifetime in perovskite films prepared with SnBr_2_ purification from 807 to 131 μs. We attribute the shorter lifetime to a greater population of free photogenerated electrons able to undergo recombination (*k*_recomb_) with the PEDOT hole polarons, which is further consistent with our discussion on the removal of SnBr_4_ trap states.^[^ ^52^, ^53^, ^54^, ^55^, ^56^, ^57^, ^59^, ^61^, ^62^ ^]^ To further verify the removal of SnBr_4_ as the origin of changes in the TAS spectra, 5% SnBr_4_ was added to the precursor solution. Consistent with our hypothesis, we note TAS of the SnBr_4_‐containing perovskites yield a decrease in Δ*OD*_max_ and longer lifetimes, closely resembling the unpurified samples (Figure S5, Supporting Information).^[^ ^50^, ^53^ ^]^

### 2.4 Effect of SnBr_4_ Removal on Photovoltaic Performance and Electronic Properties

**Figure 5:**
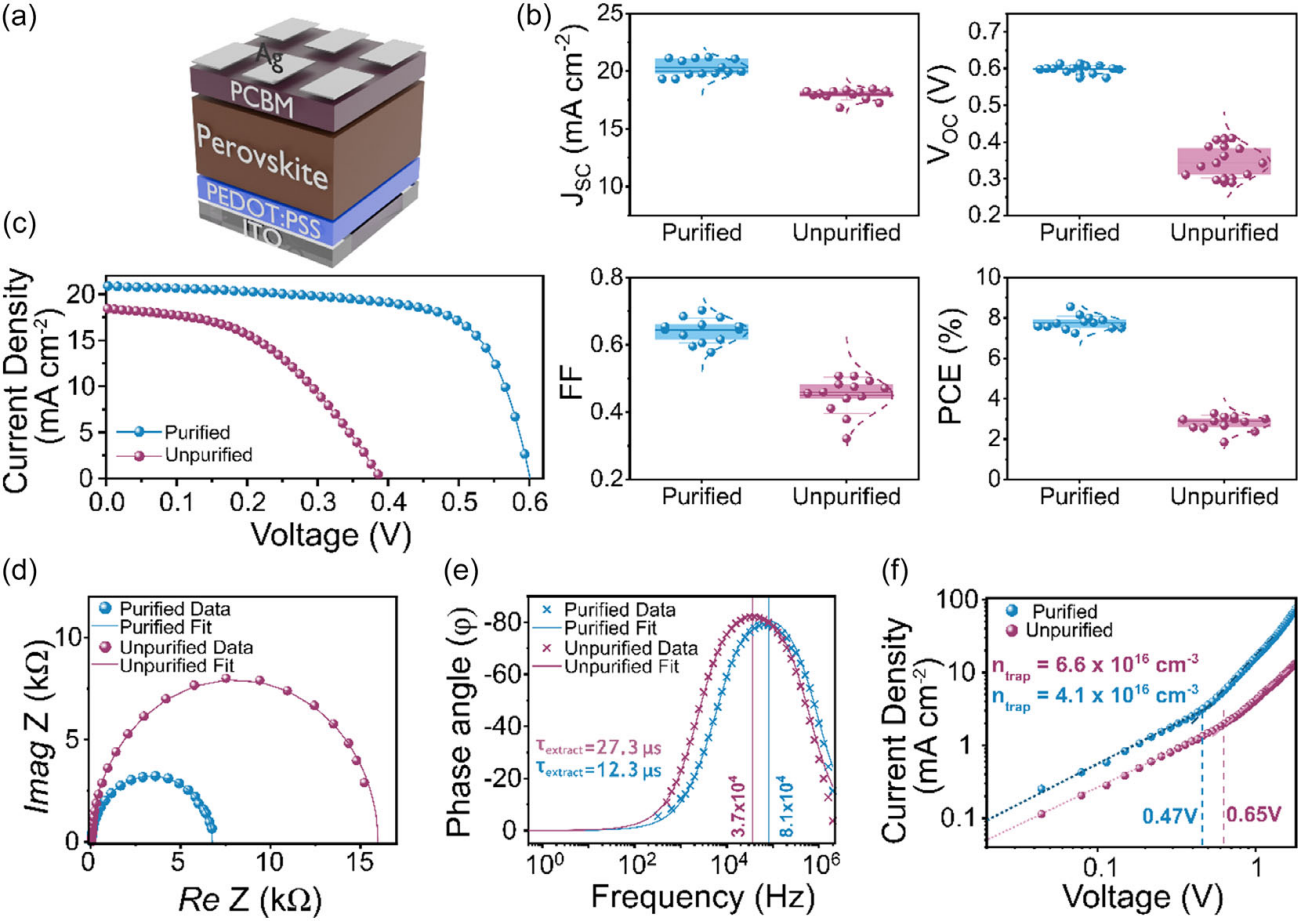
P‐I‐N solar cells with a PEA_0.2_FA_0.8_Sn(I_0.9_Br_0.1_)_3_ active layer prepared with purified and unpurified SnBr_2_. a) Schematic of the device architecture (BCP not shown). b) Statistical data of the short‐circuit current (*J*_sc_, calibrated using EQE, Figure S7b, Supporting Information), open‐circuit voltage (*V*_oc_), PCE, and FF (left to right, top to bottom). c) *JV* scans in reverse on champion pixels. d) Nyquist plot of unpurified and purified PSCs measured under light conditions at *J*_sc_. e) Frequency‐phase angle plot measured under light conditions at *J*_sc_, with the dashed line indicating the peak extraction frequency. Derived extraction times are annotated in accordance with Equation (1). f) SCLC voltage–current plot using an electron‐only device. Linear regions are fitted with dashed lines, and the vertical point of interception marks the trap‐filled voltage (*V*_TFL_).

To understand the impact of SnBr_4_ and the associated defect chemistry on photovoltaic parameters, solar cell devices were fabricated using an inverted architecture consisting of ITO/PEDOT:PSS/Sn‐perovskite/PCBM/BCP/Ag (**Figure** **5**a) (Figure S6, Supporting Information). We use this archetypal architecture as a representative configuration widely used in the literature (with an acknowledgment that current highest‐performing Sn‐PSCs now adopt ICBA/BCP), allowing us to directly investigate the impact of SnBr_4_ impurities on photovoltaic performance.^[^ ^63^, ^64^, ^65^ ^]^ The statistical distribution of the device performance is shown in Figure 5b, and *JV* curves of champion devices prepared using purified and unpurified SnBr_2_ are presented in Figure 5c. Indeed, PSCs fabricated using purified SnBr_2_ enabled a significant performance improvement from 3.3% to 8.5%, upon removal of the Sn(IV) states, consistent with our optoelectronic, morphological, and charge injection findings. The effect of SnBr_4_ removal on photovoltaic performance is further supported with a statistical improvement across all photovoltaic parameters (Figure 5b). In particular, we find that the removal of SnBr_4_ leads to significant improvements in both *V*_OC_ from 0.34 to 0.6 V and fill factor (FF) from 0.45 to 0.64 (**Table** **1**). Interestingly, these improvements exhibit similarities to our previous observations on the removal of SnI_4_ impurities within photovoltaic devices,^[^ ^9^ ^]^ as well as reported use of unpurified SnBr_2_ in the literature.^[^ ^32^ ^]^ To confirm that the presence of trace SnBr_4_ as the origin of performance losses in devices prepared without purification, additional devices were prepared with inclusion of 5% SnBr_4_ (Table 1, Figure S7, Supporting Information), corresponding closely to our calculated Sn(IV) content using TGA and UV–vis (5.6%) Consistent with our findings to this point, these devices exhibit significantly worsened performance, resembling those prepared without purification. Moreover, analogous to the unpurified devices, it is clear that devices with +5% SnBr_4_ are hindered predominantly the introduction of Sn(IV) to decrease FF and *V*_OC_, with the latter commensurate with an analogous study on iodide‐only Sn‐PSCs involving the deliberate addition of SnI_4_.^[^ ^9^ ^]^

**Table 1:** Summarized statistics for purified and unpurified devices shown in Figure 5b (15 pixels, Figure 5b) and devices with the addition of 5% SnBr_4_ to the purified SnBr_2_.

	*J*_sc_ [mA cm^−2^]	*V*_OC_ [V]	FF	PCE [%]
Unpurified	17.95 (±0.45)	0.34 (±0.04)	0.45 (±0.05)	2.79 (±0.37)
Purified	20.29 (±0.68)	0.60 (±0.01)	0.64 (±0.04)	7.75 (±0.33)
Purified + 5% SnBr_4_	16.78 (±2.58)	0.43 (±0.07)	0.47 (±0.04)	3.21 (±0.52)

Further insights into the effect of Sn(IV) trap states on the device performance were obtained using electrochemical impedance spectroscopy. We begin by obtaining Nyquist plots of devices under illumination at short‐circuit conditions (Figure 5d). In this measurement photogenerated carriers are subjected to the maximum built‐in electric field (*E*_BI_) allowing for insights into Sn(IV) trap states and their effect on the extraction dynamics of carrier within the devices. Fitting the plots, we observe a decrease in charge transfer resistance (*R*_CT_) upon SnBr_4_ removal, consistent with a more compact morphology with fewer defects as observed in the SEM (Figure 2c,d, Figure S16, Supporting Information). Further measurements on the reactance within the devices were collected to provide additional information on the effect of the trap states and interfacial resistance on the carrier extraction lifetimes. Constructing Bode plots (Figure 5e), we attribute the high‐frequency capacitance peak as the accumulation of photogenerated carriers at the interfaces of the active layer, giving a time scale for extraction. Converting the peak frequency to a lifetime using Equation (1) we obtain charge extraction lifetimes of 12.3 and 27.3 μs for the perovskite devices prepared using purified and unpurified SnBr_2_, respectively.^[^ ^66^ ^]^

(1) $$\tau =\frac{1}{2\pi f}$$

(2) $$V_{\text{TFL}}=\frac{qL^{2}N_{t}}{2\varepsilon_{r}\varepsilon_{o}}$$

(3) $$\;J=\frac{9}{8}\varepsilon_{r}\varepsilon_{o}\mu \frac{V^{2}}{L^{3}}$$

Space charge limited current (SCLC) was used to compare and estimate the trap‐state density within the perovskite active layer before and after SnBr_2_ purification (Figure 5f). To do this, electron‐only devices consisting of ITO/SnO_2_/perovskite/PCBM/Ag were prepared. From the log *JV* plot, we observe two distinct regions of current–voltage response, whereby a switch from the linear region where *J *≈ *V* ^1^ to a *V* ^2^ indicates the presence of a space‐charge limited conduction regime.^[^ ^55^, ^67^, ^68^ ^]^ Consistent with our spectroscopic findings, we note a significant decrease in the trap‐filled limited voltage (*V*_TFL_), the point at which the current density transitions from linear to proportional to *V* ^2^. As such, a decrease in the voltage in which all traps are filled is indicative of a lower density of trap states within the perovskite layer as per Equation (2).^[^ ^67^, ^68^, ^69^ ^]^ To convert this to a numerical estimate, we next determine the dielectric constants of the perovskite layers (180 nm thickness confirmed with SEM, Supplementary Figure S16, Supporting Information) to be 25.5 and 28.2 with and without purification, respectively, via capacitance frequency (*C*–*f*) measurements of the geometric capacitance (Note S1, Figure S8, Supporting Information). Using these values we can estimate trap‐state densities (*N*_t_) of 6.6 × 10^16^ cm^−3^ and 4.1 × 10^16^ cm^−3^ before and after purification to remove SnBr_4_, respectively (Note S1, Figure S9 and S10, Supporting Information). Considering the nature of the purification to reduce the content of Sn(IV) states, such a reduction in trap density is likely to arise via the removal of deep trap states that are responsible for a large loss in photogenerated carriers and consequent reductions in photovoltaic performance.^[^ ^70^ ^]^ Therefore, this ≈40% decrease in trap density is consistent with both our spectroscopic and structural findings and helps to rationalize the direct consequence of even trace SnBr_4_ inclusion on photovoltaic performance. Likewise, estimation of the mobility using the Mott–Guerney law (Equation (3)) applied to the space‐charge limited conduction region reveals over a fivefold increase in electron mobility (μ_e_) from 1.1 to 6.7 × 10^−5^ cm^2^V^−1^s^−1^ upon removing SnBr_4_ from the precursor (Note S1, Figure S11, Supporting Information). An increase in mobility upon removal of Sn^4+^ states is in good agreement with our charge extraction lifetimes under illumination and highlights the impact of SnBr_4_ formation on the optoelectronic performance. Indeed, the reduced mobility of carriers within the perovskite leads to poorer conductivity in the ohmic region of SCLC and higher series resistance (*R*_s_) when comparing dark *JV* data of devices (Note S1, Figure S12, Supporting Information).

To this point we have demonstrated that even at low concentrations, the inclusion of SnBr_4_ has dramatic effects on the perovskite crystal structure, morphology, and carrier dynamics. These effects in turn have a significant impact in facilitating nonradiative recombination, limiting carrier mobility, and significantly reducing the performance of photovoltaic devices. These findings on the impact of initial trace SnBr_4_ species within SnBr_2_ can rationalize why the use of SnBr_2_ within the perovskite precursor has not yet been extensively adopted in contrast to PbBr_2_ in analogous Pb‐systems.

Having identified the dramatic effect of SnBr_4_ and the ability to nullify the adverse effects on performance through various purification mechanisms, we next discuss the opportunities afforded by the availability of tin salts (SnBr_2_) as a high‐quality bromide source for compositional engineering (e.g., tuning halide composition in ABX_3_ perovskites). Indeed, introducing Br^−^ anions via a purified tin salt offers two key advantages over the organohalide. Firstly, by introducing SnBr_2_ as a tin salt the stoichiometric requirement of SnI_2_, a highly unstable precursor salt, can be minimized. This stems from the unique iodine chemistry associated with SnI_2_ and the higher oxidation potential of SnBr_2_, as will be shortly discussed.^[^ ^26^ ^]^ Secondly, the introduction of SnBr_2_ becomes unavoidable in the preparation of perovskite compositions with bromide stoichiometries exceeding the 1:2 ratio (33%) from the use of organohalides alone. In such cases, we have shown the need for minimizing SnBr_4_ is essential and can be achieved simply via means of purifying starting materials.

(4) $$2{\text{SnX}}_{2}+O_{2}\to {\text{SnO}}_{2}+{\text{SnX}}_{4}\;\;\left(\text{X=Br, I}\right)$$

(5) $${\;C}_{7}H_{8}{+\text{Br}}_{2}\to C_{7}H_{7}\text{Br+HBr}$$

**Figure 6:**
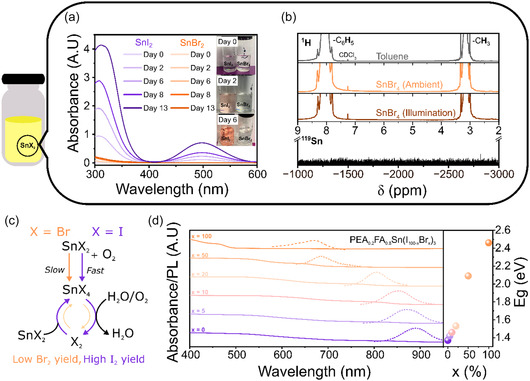
Comparison of the chemical stability between SnBr_2_ and SnI_2_. a) UV–visible absorbance spectrum of 1.5 mM SnI_2_ and SnBr_2_ dissolved in toluene taken over a 13 d interval. The inset shows the colors of the solutions between 1–6 d. b) NMR spectra of: (gray) ^1^H spectra of toluene reference sample, (orange) ^1^H spectra of 1.5 mM SnBr_4_ in toluene following 3 weeks exposure to ambient conditions, (brown) ^1^H NMR spectra of 40 mM SnBr_4_ of toluene left for 1 week in ambient conditions followed by 12 h in ambient conditions and illumination (40 W) bulb, and (black) ^119^Sn spectra of SnBr_4_ left in ambient conditions over 1 week and illuminated for 12 h. All samples were left for an additional 5 min under 1 sun illumination (100 mW cm^−2^). An additional 100 μL of CDCl_3_ was added to each solution to provide an NMR lock signal and a reference signal *δ*CDCl_3_ = 7.26 ppm. c) Proposed reaction scheme of degradation of SnX_2_ with oxygen. d) Left: UV–visible absorbance (straight line) and PL emission (dashed line) for varied compositions of *x*(%) in PEA_0.2_FA_0.8_Sn(I_1‐*x*_Br_*x*_)_3_ films. Right: Derived bandgaps from UV–visible absorbance. Tauc plots are given in Figure S15, Supporting Information.

Addressing first the substitution of SnI_2_ with SnBr_2_ we prepare a simple experiment whereby 1.5 mM solutions of SnI_2_ and SnBr_2_ were left in toluene in ambient conditions to characterize the nature and rate of any degradation products upon exposure to ambient environmental conditions. Absorbance spectra were collected of the two solutions at regular time intervals (Figure S13, Note S2, Supporting Information). The spectra collected show a notable difference between the SnI_2_ and SnBr_2_ chemical decomposition during ageing (**Figure** **6**a). As per previous reports, in the case of SnI_2_ a dramatic increase in absorbance is observed at 330 and 500 nm, consistent with the mechanism of I_2_ formation occurring via an SnI_4_ intermediate state.^[^ ^9^ ^]^ In contrast, SnBr_2_ exhibits a gradual increase at wavelengths <330 nm which we attribute to SnBr_4_ in accordance with our previous absorbance spectra (Figure 1a). As such, we find that the reaction with air (Equation (4)) occurs in both SnI_2_ and SnBr_2_, however on significantly slower timescales in the case of the latter. We next look to probe if the evolution of the molecular halide (Br_2_) is partly responsible for the slower oxidation. The combination of its volatility, reactivity, and lack of infrared modes makes the identification of bromine as a decomposition product challenging and has consequently resulted in little discussion within perovskite literature. In this work, benzyl bromide was used as a molecular probe for Br_2_. Benzyl bromide forms rapidly from the reaction between molecular bromine and toluene under illumination, in a radical‐based reaction known as benzylic bromination (Equation (5)), Figure S14, Supporting Information).^[^ ^71^, ^72^ ^]^ To probe the formation of bromine, SnBr_4_ was again aged in toluene and left for 1 week in ambient conditions before being exposed to illumination (40 W tungsten bulb) for 12 h. ^1^H NMR of the solution failed to show any new peaks at chemical shifts resembling benzyl bromide (*δ*_H_(‐CH_3_) 4.44 ppm), which we use here as a Br_2_ marker.^[^ ^73^, ^74^ ^]^ Furthermore, no benzyl bromide peaks were observed even upon the direct addition of 10% water, a known prerequisite for the analogous chemistry of iodine formation.^[^ ^9^ ^]^ This result was further confirmed using ^119^Sn NMR showing only a single peak we previously attribute to SnBr_4_ in a toluene /CDCl_3_ cosolvent (Figure 6b). This is consistent with the known tendency of SnBr_4_ to form the hydrate SnBr_4_·(H_2_O)_2_, rather than undergo complete hydrolyzation to HBr and subsequent oxidation to Br_2_. As such we expect the catastrophic catalytic degradation cycle (Figure 6c) of the Sn^4+^ salt to a molecular halide to not be present within tin. Based on these findings, we highlight SnBr_2_ to be a more stable tin precursor to SnI_2_.

In a second opportunity afforded by understanding the chemistry of SnBr_2_ as a precursor, we demonstrate the creation of bromide‐rich perovskite compositions available when using a low starting SnBr_4_ concentration. Notably, in these cases introduction of bromide ions via organobromide salts is insufficient to meet stoichiometric requirements and thus, the need for understanding and managing SnBr_4_ chemistry becomes unavoidable. We demonstrate that by varying the PEA_0.2_FA_0.8_Pb(Br_x_I_1‐*x*_)_3_ ratio from *x* = 0 to 1 a wide range of bandgaps can be produced from 1.4 to 2.4 eV (Figure 6d, Figure S15, Supporting Information). To this point, we show that the introduction of Br^−^ ions (*x* = 0.1) in the form of SnBr_2_ can have benefits over the use of SnI_2_ by virtue of its lower tendency to form an oxidizing molecular halogen.^[^ ^29^, ^34^ ^]^ This result highlights new opportunities to reduce the concentration of unstable SnI_2_ within the starting precursor.

## 3 Conclusion

In conclusion, we have shown that as‐received commercial SnBr_2_ contains a significant percentage of SnBr_4_ impurities which we demonstrate have dramatic adverse effects on the intrinsic and optoelectronic material properties. These findings may explain the avoidance of SnBr_2_ salts as a precursor in Sn‐perovskites and a reliance on organobromides. Even 5% SnBr_4_ within SnBr_2_ starting material introduces lattice strain and crystallographic disorder, effecting the morphology and introducing defects. Likewise, spectroscopic techniques reveal even trace SnBr_4_ is sufficient to significantly reduce emission intensity and lower the yield of holes removed from the cell. A range of electronic and optoelectronic techniques directly probed SnBr_4_‐based traps, showing increased trap density and reduced electron mobility, which slows carrier extraction and promotes recombination, reducing device performance.

Previously these implications on performance have mandated the use of organobromide salts, ignoring the potential benefits of using SnBr_2_ to substitute unstable SnI_2_. Purification techniques such as sublimation, used here, significantly reduce the concentration of SnBr_4_, dramatically improving the optoelectronic properties of Br‐containing Sn‐perovskite films. Crucially, we demonstrate that following sufficient purification, SnBr_2_ exhibits superior intrinsic chemical stability to SnI_2_, making it a preferred tin salt. Comparison of halide chemistry shows that whereas SnI_2_ undergoes rapid decomposition accelerated by the formation of I_2_, analogous chemistry cannot occur in the SnBr_2_ salt. Overall, these results highlight both the negative impact of SnBr_4_ impurities and the opportunities afforded upon mitigating the challenges associated with SnBr_4_.

## Supporting Information

Supporting Information is available from the Wiley Online Library or from the author.

## Conflict of Interest

The authors declare no conflict of interest.

## Supporting information

Supplementary Material

## Data Availability

The data that support the findings of this study are available from the corresponding author upon reasonable request.
