# Supplementary material for: Understanding and Overcoming the Fundamental Chemical and Electronic Challenges of SnBr4 Impurities in Tin Perovskite Solar Cells
Source: Small Sci. 2025 Nov 11;5(12):e202500426. doi: 10.1002/smsc.202500426 (PMC12697828; doi:10.1002/smsc.202500426)
Supplement: Supplementary file 1 — Supplementary Material [file SMSC-5-e202500426-s001.pdf]

Understanding and overcoming the fundamental chemical and electronic challenges of $SnBr_4$ impurities in tin perovskite solar cells

Amanz Azaden†[1], Thomas Webb†[1], Polina Jacoutot[1], Harry Spear[1], Robert Palgrave*[2], Saif A Haque*[1]

[1]Molecular Sciences Research Hub, Department of Chemistry, Imperial College London, White City, London, W12 0BZ

[2] Department of Chemistry, University College London, 20 Gordon St, London WC1H 0AJ

† These authors contributed equally

Corresponding authors: s.a.haque@imperial.ac.uk & r.palgrave@ucl.ac.uk

Experimental procedure:

Formamidinium iodide (FAI) and phenethylammonium iodide (PEAI) were purchased from Greatcell Solar. Tin(II) iodide (SnI2, 99.99% following sublimation treatment), Sn metal (99%), Tin(II) Bromide ($SnBr_2$), Tin(IV) bromide ($SnBr_4$) and tin(II) fluoride (SnF2, 99%) were purchased from Sigma Aldrich. All solvents were purchased from Acros Organics.

$SnBr_2$ precursor purification

As received $SnBr_2$ packed under N_2 was opened in an acrylic glovebox 1ppm> and placed inside a covered Petri dish, which was then put onto a hot plate and heated at 120 °C for 1 h to eliminate $SnBr_4$ via sublimation. After the treatment, $SnBr_2$ was kept under dark conditions under N_2 in a clean vial for future use.

Thin film fabrication

Microscope glass substrates (Fisher) were cut to ≈ 1.3 cm × 1.3 cm and sequentially sonicated in soapy water, deionised water, acetone, and isopropanol alcohol. Prior to deposition, the glass slides were surface-treated using ultraviolet-ozone surface treatment for 15 min. They were immediately transferred to an MBraun N_2 GB (ppm O2 0.5ppm>). A perovskite precursor (either $PEA_{0.2}FA_{0.8}Sn(I_{0.9}Br_{0.1})_3$ or $PEA_{0.2}FA_{0.8}SnI_3$)*were prepared in a glovebox from PEAI, FAI, $SnBr_2$, SnI2 and SnF2 powders in a mixed solvent of 4:1 v/v mixture of DMF and DMSO (both ultradry and bottled with molecular sieves). The precursor solution was stirred and heated at 50 °C for 1 h before use. The solutions were then filtered (0.22 µm, PTFE) and deposited onto the substrate via a one-step spin-coating procedure (4000 rpm for 20 s, 700 µl of dry diethyl ether dropped in the middle of the substrate at 9 s, followed by annealing at 70 °C for 20 min.*

Device fabrication

ITO (15 Ohm sqm^{-2})-coated glass were prepared identically to the glass substrates (vide supra). PEDOT:PSS was then filtered and spin-coated onto the ITO at 5000 rpm with 2000 rpm acceleration for 30 s under ambient conditions, and subsequently annealed at 140 °C for 20 min. Perovskite films were deposited identically to the thin films. PCBM (15 mg mL−1 in chlorobenzene) was subsequently deposited by spin-

coating at 2000 rpm for 30 s, followed by dynamic spin-coating of bathocuproine (BCP, 0.5 mg mL^{-1} in IPA) atop the PCBM at 5000 rpm for 20 s. A 100 nm Ag electrode was then deposited via thermal evaporation under a vacuum of 10^{-6} mbar to produce devices with an active area of 0.045 cm^2.

Device characterisation

Current-voltage (J-V) characteristics were obtained using a Keithley 2400 source meter using a scan rate of 50 mV s−1 under forward bias conditions. The devices were subjected to 100 mW cm−2 of illumination using a 150 W Xenon lamp with an AM 1.5 filter (ScienceTech). Light intensity was calibrated using an independent calibrated silicon photodiode and checked every hour. Devices were kept under inert conditions during the measurements by loading them in a N_2 glovebox into a sealed measuring chamber.

UV-Visible spectroscopy

UV-Visible spectroscopy measurements were performed with a Shimadzu UV-2600 integrating-sphere spectrophotometer. Scan ranges of 300 nm to 1000 nm were used for steady state scans. Thin films were mounted onto an aperture-holder for equal area irradiation, and solution measurements were done using a 3mL quartz cuvette. Beer lambert calculations were undertaken by dissolving $SnBr_4$ (0.57-0.06mM) and measuring the absorbance to obtain the molar extinction coefficient.

***Time Correlated single photon counting (TCSPC**)*

TCSPC plots were obtained with a Horiba Deltaflex Modular Fluorescence Lifetime setup fitted with a PPD 900 detector. The excitation wavelength was chosen as 404 nm and applied via a nanoled (Model N-07; repetition rate: 1 MHz; pulse duration < 200 ps). Due to sample instability, perovskite films deposited on glass were measured inside a quartz cuvette.

Steady state Photoluminescence spectroscopy

Steady state PL was collected under inert conditions using a sealed quartz cuvette. Excitation was achieved with a 5 mW diode laser at a wavelength of 404 nm. The emission was collected at an angle of 90 ^{o} and collected through a collimator into a fibre coupled Thorlabs CCS175 500-1000 nm spectrometer.

Transient Absorbance Spectroscopy

Samples for TAS were prepared using a glass/mp-TiO_2/Perovskite/PEDOT architecture. An mp-TiO_2 electron sync was prepared via diluting TiO_2 paste (Dyecell) in ethanol in a 7:2 ratio (Ethanol:TiO_2) before stirring overnight. The perovskite layer was prepared as previously stated. For the PEDOT layer HTL solar 3 (Osilla) was chosen to provide solvent orthogonality.

Samples for microsecond transient absorption spectroscopy measurements were stored in a nitrogen-filled glovebox and taken in a sealed quartz cuvette for measurement. Excitation was achieved with Nd:YAG laser (Opolette UX tunable OPO laser). The laser fluence at the point of measurement was 120 µJ cm^{-2}. The transient changes in the absorption of the sample were captured with a 100 W tungsten lamp (Bentham IL 1) on an orthogonal optical axis to the excitation beam. The probe wavelength was adjusted by a monochromator and set to 1600 nm . This probing beam was detected by an InGaAs photodiode (Hamatsu Photonics) before being filtered and amplified (Costronics Electronics) and finally interpreted by a digital

oscilloscope (Tektronics DPO3012). TAS decays were modelled with a stretched exponential function: $\Delta OD \propto \exp[-(tto)a]$. Given the complex decay dynamics, an approximate lifetime, τ_{rec}, is assigned as the time taken for ΔOD to reach 50% of its original value.

NMR spectroscopy

Samples for NMR were dissolved in toluene with 10% $CDCl_3$ to allow for lock and shim of the deuterium signal. Samples were were measured on a Bruker Avance III HD 400 MHz NMR, collected with topspin 3.2 software and processed in Mestrenova.

EIS

Electrochemical impedance spectroscopy was measured using a Gamry 1010E digital potentiostat. Samples were measured under N_2 using a sealed chamber and multiplexer.

X-ray diffraction

Diffraction patterns for perovskite films were obtained using a PANalytical X'Pert Pro MRD diffractometer, where incident X-rays originated from a Cu K-α X-ray source (λ = 1.54 Å) at 40 kV and 40 mA. The diffraction patterns were obtained over the 2ϑ range 5° - 35° in steps of 0.02°, with the samples rotated during measurement. The FWHM was computed using area analysis on Origin Pro to input into the Scherrer equation for crystallite size calculations.

Thermogravimetric analysis

TGA measurements were conducted with a Mettler Toledo Thermogravimetric analyser under an N2 atmosphere at a 5 °C/min heating rate.

Scanning electron microscopy

SEM images were taken with an Zeiss Field Emission Scanning Electron Microscope used at 5 kV 5mm WD and fitted with an In-Lens detector. Prior to image acquisition, analysed films were unavoidably exposed to air (~1 min) and coated with 10 nm of Cr via sputtering.

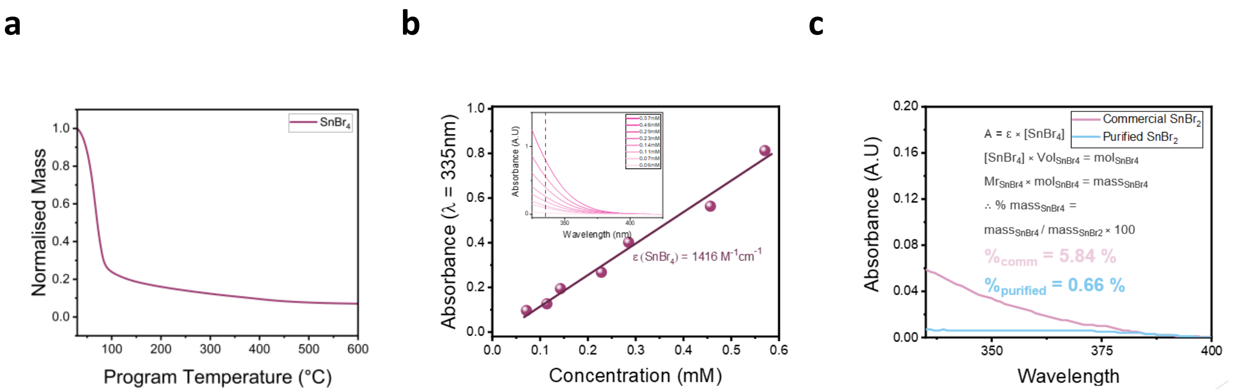

Supplementary Figure S1: $SnBr_4$ powder investigation: a) TGA of $SnBr_4$. **b)** UV-visible absorbance of tin bromide powders. Left: concentration absorbance plot collected for a wavelength of 335 nm of $SnBr_4$, fitted to a straight line to compute the molar extinction coefficient with a cuvette of path length (l) 1 cm for A = εcl. Inset shows the absorbance plots of varied concentration, and the dashed line represents the chosen wavelength of 335 nm. **c)** absorbance of $SnBr_2$ solution ~1 mg/mL for a purified and commercial $SnBr_2$ sample.

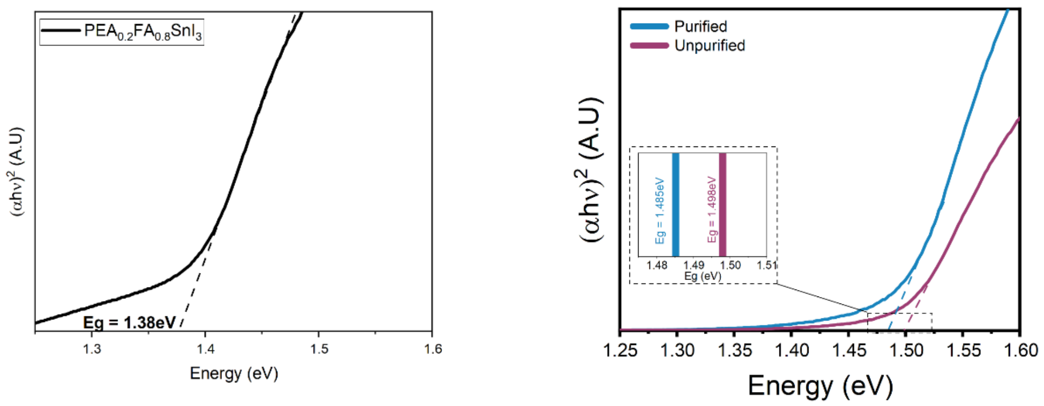

Supplementary Figure S2: Band gap derived from UV vis absorbance plots – Left: Tauc plot of a $PEA_{0.2}FA_{0.8}SnI_3$ thin film. Right- Tauc plots of purified and unpurified $PEA_{0.2}FA_{0.8}Sn(I_{0.9}Br_{0.1})_3$ films.

Table S1: Calculated lifetime fits to a biexponential decay for PEA0.2FA0.8Sn(I0.9Br0.1)3 films. The Tav represents a weighted average.

Sample	A_1	t_1 (ns)	A_2	t_2 (ns)	t_{av} (ns)	R^2
Commercial	0.81	0.38	0.18	1.01	0.61	0.99
Purified	0.97	9.04	0.06	18.84	10.16	0.99
Purified + 5% $SnBr_4$	0.87	0.50	0.23	0.50	0.50	0.99

Equation S1: biexponential decay fitting used.

$$y = y_0 + A_1 e^{-x/t_1} + A_2 e^{-x/t_2}$$

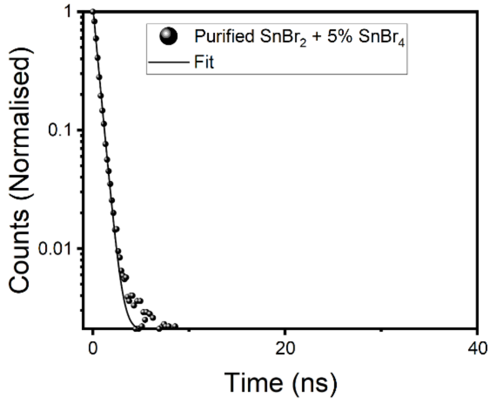

Supplementary Figure S3: Time correlated single photon counting (TCSPC) of a $PEA_{0.2}FA_{0.8}Sn(I_{0.9}Br_{0.1})_3$ film fabricated using a precursor solution containing purified $SnBr_2$ with 5% addition of $SnBr_4$.

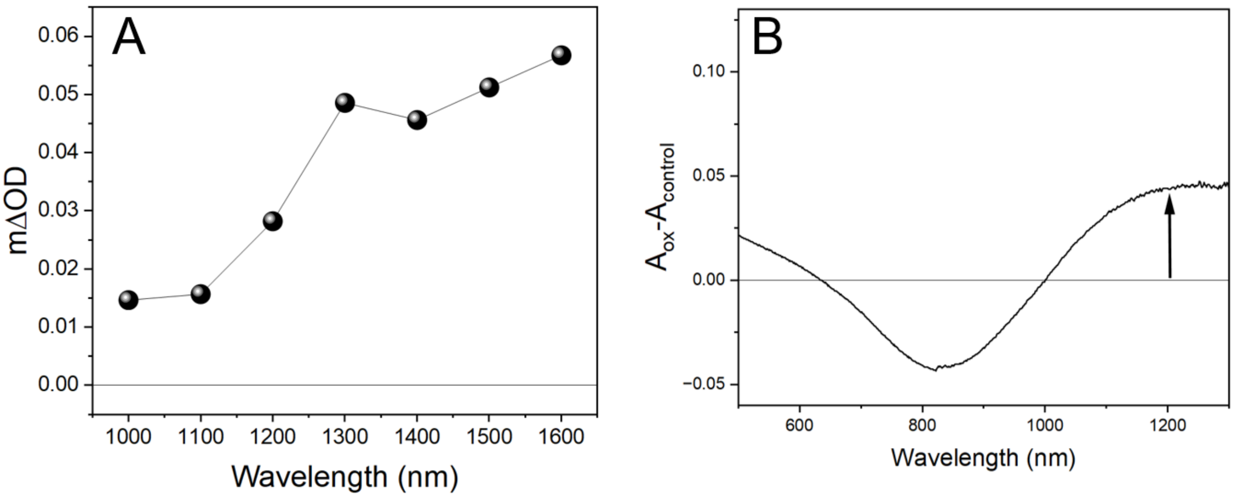

Supplementary Figure S4: Comparison of ΔOD and changes in PEDOT upon oxidation. A) Plot of OD_{max} against wavelength showing an increase towards the near IR region of the spectra. B) Comparison of PEDOT after chemical oxidation with $AgNO_3$ as an oxidizing agent showing the changes upon chemical oxidation. Spectra shows a broad increase towards the near IR region consistent with the ΔOD spectra.

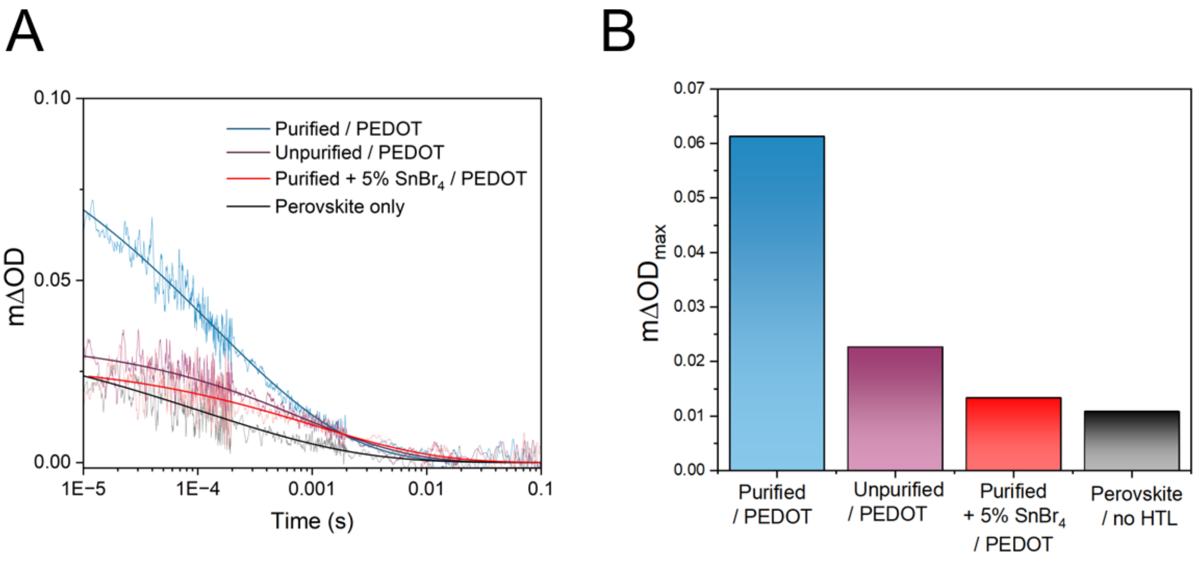

Supplementary Figure S5: TAS data of the hole bipolaron in PEDOT with purification, without purification and with 5 % $SnBr_4$ deliberately added to the precursor A) fitted decay data with time and B) comparison of maximum ΔOD, corresponding to hole yield.

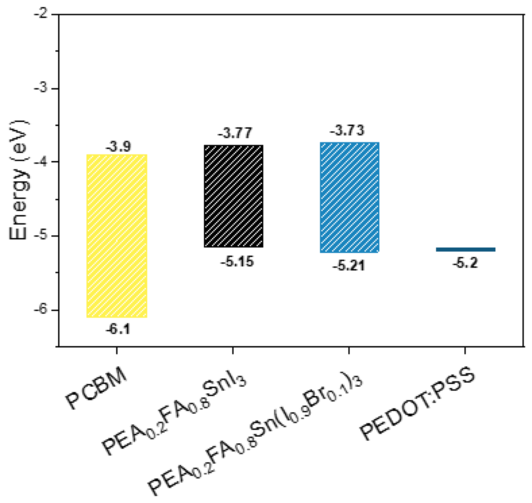

Supplementary Figure S6: Energy diagram of perovskite materials and associated CTLs for the device fabrication. Optical band gap was determined from the derived Tauc plots of UV vis absorbance (Figure S2). The position of PEDOT:PSS was taken from reference[14].

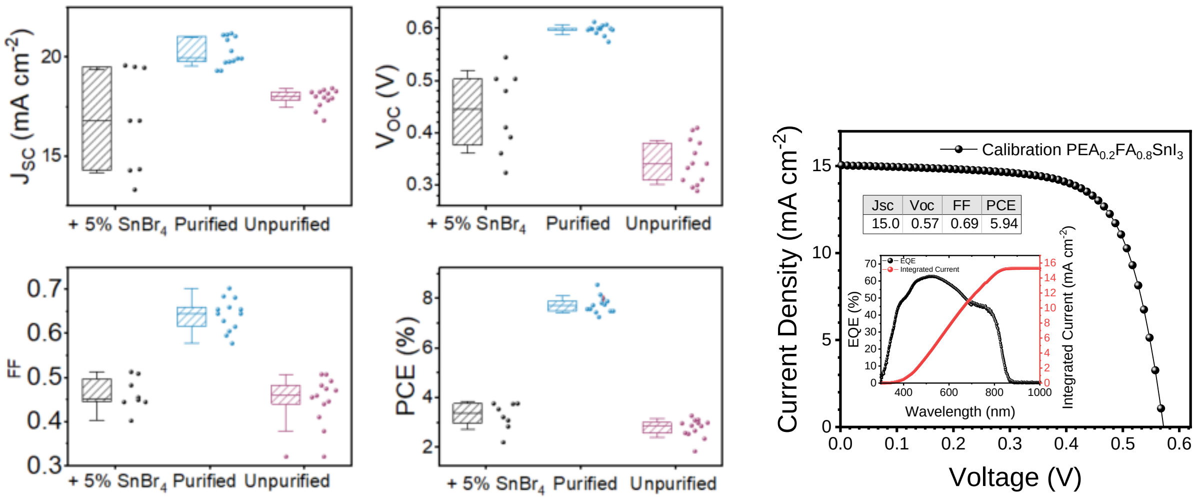

Supplementary Figure S7: P-I-N solar cells with a) a $PEA_{0.2}FA_{0.8}Sn(I_{0.9}Br_{0.1})_3$ active layer prepared with purified +5% $SnBr_4$, purified and unpurified $SnBr_2$. Statistical data of the short circuit current (J_{sc}), open circuit voltage (V_{oc}), power conversion efficiency (PCE) and Fill factor (FF). b) A calibration for the current using EQE on an equivalent device structure employing the archetypal $PEA_{0.2}FA_{0.8}SnI_3$ as the absorber.

Devices with added $SnBr_4$ exhibit photovoltaic parameters (PCE 3.2%, VOC 0.43 V, FF 0.47) closely resembling those of unpurified devices (2.8%, 0.34 V, 0.45), confirming that $SnBr_4$ impurities are responsible for the degraded performance.

Supplementary Note 1

Estimation of dielectric constant (ε), electron mobility (μ_e) and trap-state density (n_t)

To estimate and compare the relative number of traps between perovskite films without purification and with purification, where $SnBr_4$ is removed, we first measure the capacitance-frequency (*C*-*f*) characteristics of devices under illumination. From the plot we attain values for the geometric capacitance (C_{Geo}) corresponding to the capacitance of the bulk perovskite layer, given in Supplementary Table S2.

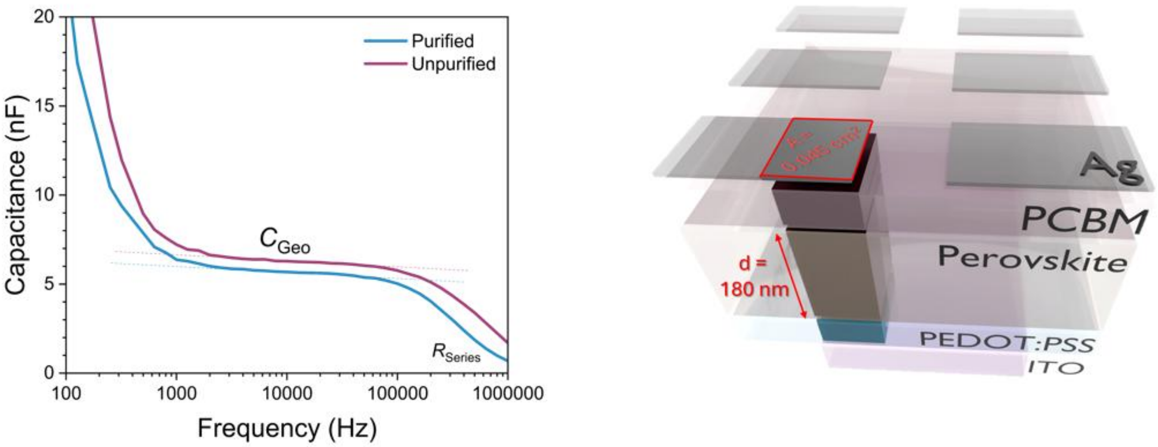

Supplementary Figure S8: Capacitance-frequency plots (left) and schematic of active area and perovskite thickness (right)

Using the geometric capacitance of the perovskite layer obtained from the *Cf* measurements, Equation Sx allows for the determination of the dielectric constant (ε_r). From SEM (supplementary figure S16) we record a perovskite thickness of 180 nm and a pixel overlap area (A) of 0.045 cm^2.

$$C = \frac{\varepsilon_0 \varepsilon_r A}{d} \text{ Equation S2}$$

$$V_{TFL} = \frac{qL^2 N_t}{2\varepsilon_r \varepsilon_o} \text{ Equation S3}$$

$$N_t = \frac{2\varepsilon_r \varepsilon_o V_{TFL}}{qL^2} \text{ Equation S4}$$

Using these values of the extinction coefficient we next look to attain information from SCLC measurements using electron only devices. The point at which the current-voltage response deviates from a linear dependency in the ohmic region ($J \propto V$) to the square of the voltage ($J \propto V^2$) in the Child region is known as the trapped filled limiting voltage (V_{TFL}) and corresponds to the voltage at which conductivity is no-longer limited by traps. The V_{TFL} can then be related to the trap density within the material via the Mott-Guerney equation. To ensure the SCLC collected is well-behaved we fit the gradient of the semi-log plot before and after the V_{TFL} voltage giving *n* values of 0.95 (UP), 1.06 (P) and 1.95 (UP), 2.28 (P) for the $J \propto V$ and $J \propto V^2$ regions, respectively. This indicates that sample does go through a space-charge limited conduction regime enabling insights into the comparative conduction properties and effect of $SnBr_4$ removal on the conductivity properties.

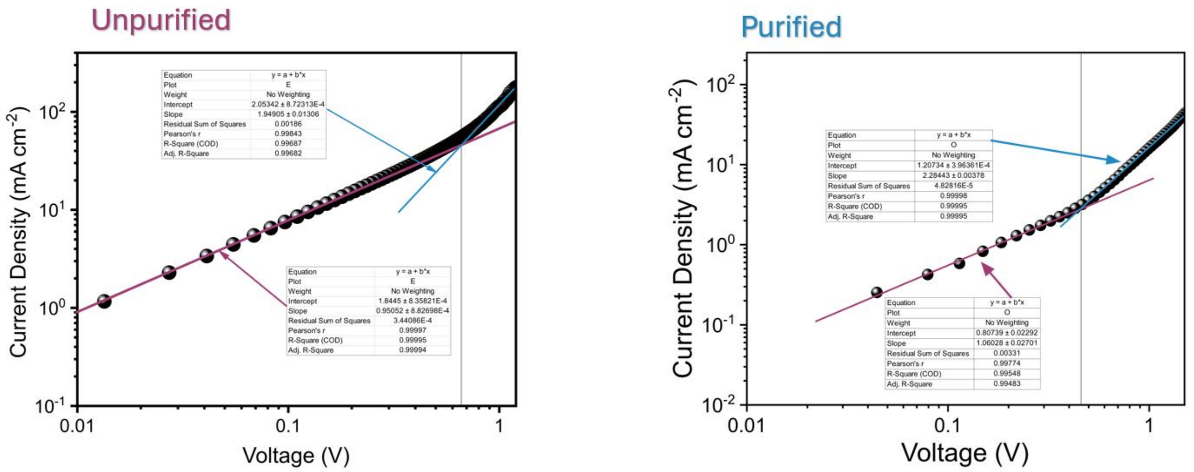

Supplementary Figure S9: log-*JV* plots of SCLC devices showing the gradient transition from a $J \propto V$ regime to $J \propto V^2$ at the V_{TFL} for SCLC devices prepared using unpurified and purified $SnBr_2$.

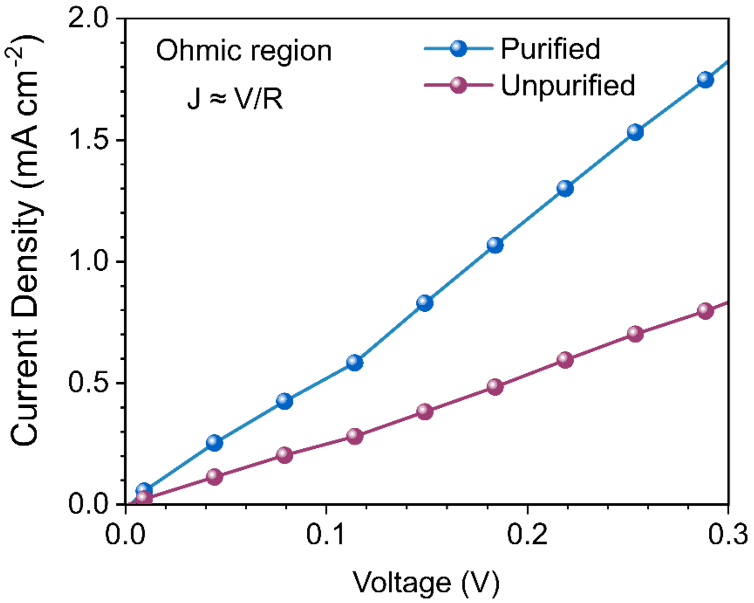

Supplementary Figure S10: Ohmic region ($J \propto V$) of SCLC data showing lower resistivity and a higher current density in perovskites prepared with purified $SnBr_2$.

Supplementary Table 2

Sample	C_{Geo} (nF)	ε_r	N_t (cm^{-3})

Unpurified $SnBr_2$	6.25	28.2	6.6 x10^{16}
Purified $SnBr_2$	5.65	25.5	4.1 x10^{16}

Using the calculated dielectric constants, measured thickness (L) of perovskite in the SCLC device, and area (0.045 cm^2) we estimate trap state densities (N_t) of 6.6 x10^{16} and 4.1 x10^{16} for perovskite films prepared without and with $SnBr_4$ purification.

Determination of electron-mobility (μ_e)

We next estimate the effect of $SnBr_4$ and associated trap-states removal on the electron mobility. Under an SCLC regime where the voltage is proportional to the square of the current ($J \propto V^2$) the current voltage characteristics can be described using the Mott-Guerney equation (below), provided charge injection into the perovskite is Ohmic:

$$J = \frac{9}{8}\varepsilon_r\varepsilon_o\mu\frac{V^2}{d^3} \text{ Equation S5}$$

$$\frac{d(J)}{d(V^2)} = \frac{9}{8}\varepsilon_r\varepsilon_0\frac{\mu_e}{d^3} \text{ Equation S6}$$

Plotting the current density against the square of the voltage and finding the gradient within the space-charge limited regime allows determination of the electron mobility provided the permittivity ($\varepsilon_r\varepsilon_0$) and thickness (d) are known (Supplementary Figure SX). Using the previously obtained values we estimate the mobilities of holes through the perovskite as 1.1 x10^{-5} $cm^2V^{-1}s^{-1}$ without purification and 6.7 x10^{-5} $cm^2V^{-1}s^{-1}$ following purification.

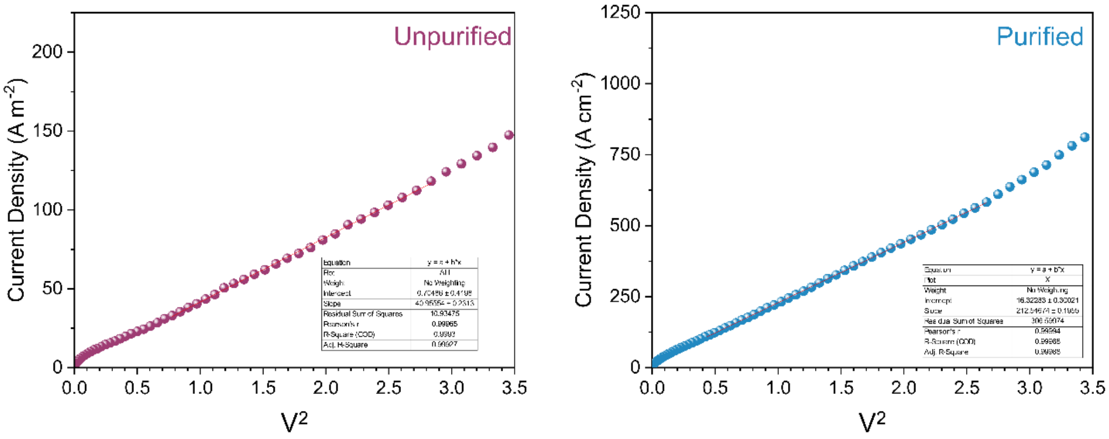

Supplementary Figure S11: Plot of J against V^2 . Gradient taken in the linear (SCLC) region to estimate and compare the electron mobilities.

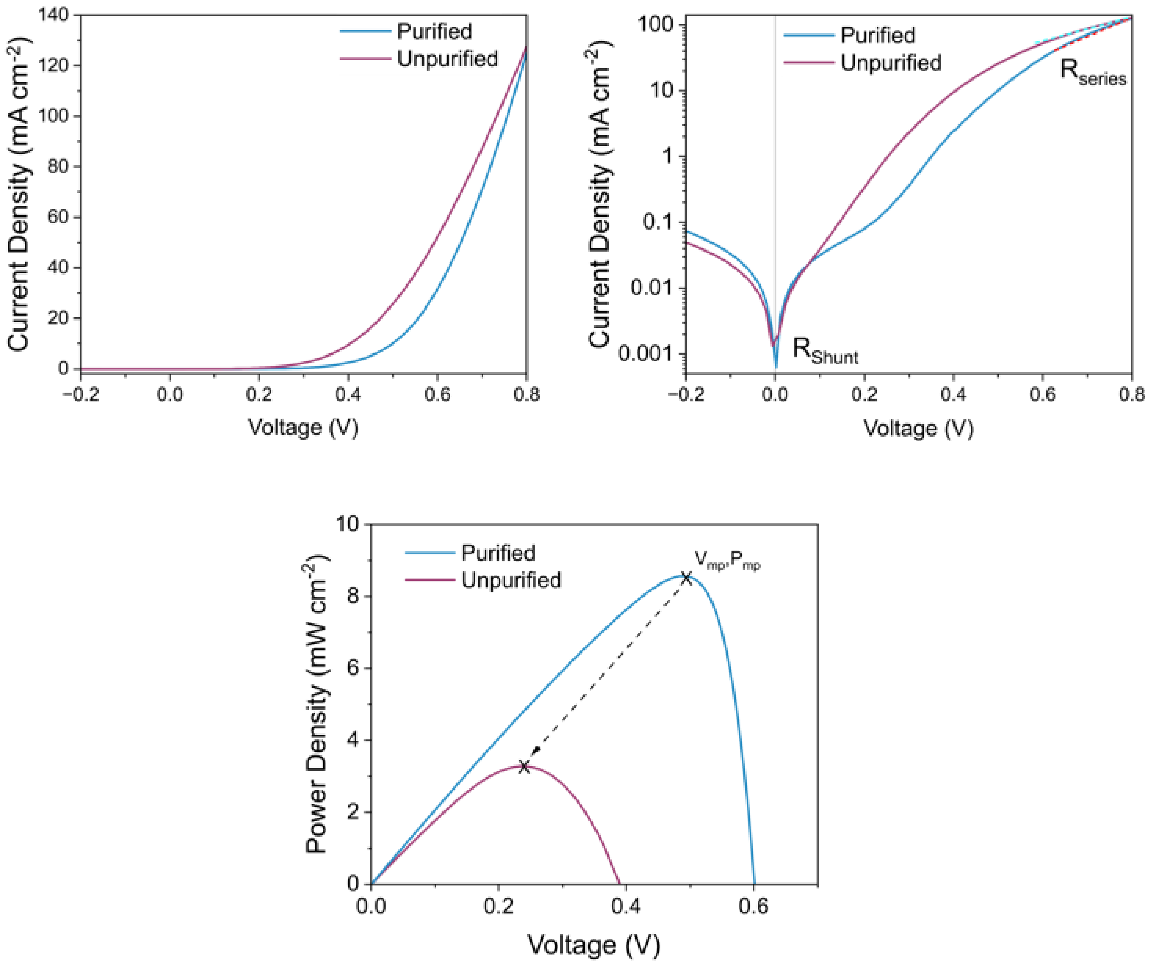

Supplementary Figure S12. (**Top left**) Dark *JV* data of devices prepared with and without purification showing greater rectifying characteristics following purification. (**Top right**) Semi-log plot of dark *JV* data where the shunt resistance is increased with purification and series resistance lowered. (**Bottom**) Power-voltage curves of champion devices with and without purification. Where the maximum power point position can be used as an indicator of series resistance as follows;

Equation S7: $P'_{max} = I_{max} V_{max} – I_{max}V_{max}$

Equation S8: $I_{max} = V_{max}/R_{max}$

Equation S9: $P'_{max} = I_{max}V_{max} - I^2_{max}R_s$

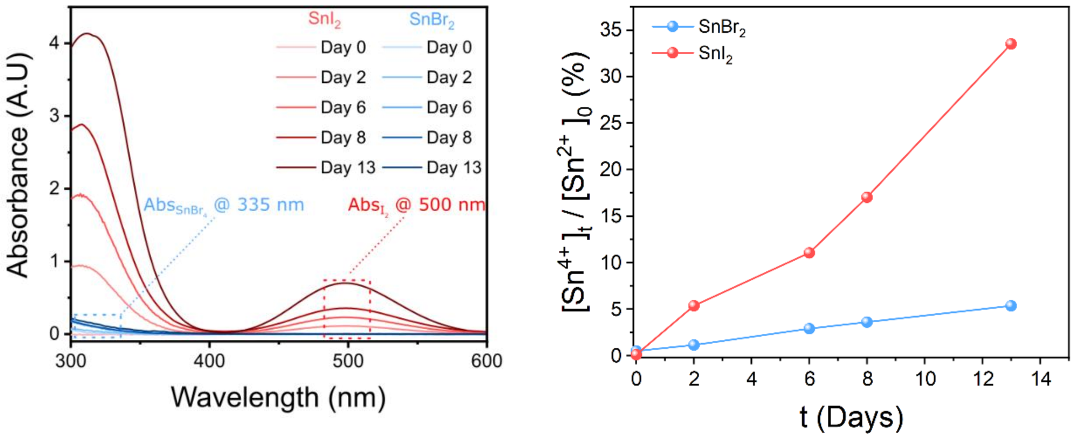

Supplementary Figure S13: Left) Absorbance spectrum of 1mM SnBr2 and SnI2 in toluene as a function of time. Right) Computed Sn(IV) content as a function of time.

Supplementary Note 2: Determination of percentage of Sn(II) oxidised to Sn(IV): Absorbance of 1mM SnI2 and 1mM SnBr2 (both purified) were aged for 13 days. The Sn(II) content in these precursors was assumed to be 100%. SnBr4 evolution was tracked at 335 nm as a function of time and the molarity was calculated using A = εcl. The ratio was determined by dividing these two molarities. For the SnI2, it has been documented that over timescales of 12 hrs, SnI4 decomposes further into I2.

$$2\ ASnI_3 + O_2 \rightarrow SnI_4 + SnO_2 + 2\ AI$$

$$SnI_4 + 2H_2O \rightarrow 4HI + SnO_2$$

$$4\ HI + O_2 \rightarrow 2I_2 + 2H_2O$$

We therefore assume that all formed I_2 is stoichiometrically in a 2:1 ratio I2:SnI_4, and use this to calculate how much SnI_4 was converted from SnI_2 and furthermore calculate the ratio of Sn(II) to Sn(IV).

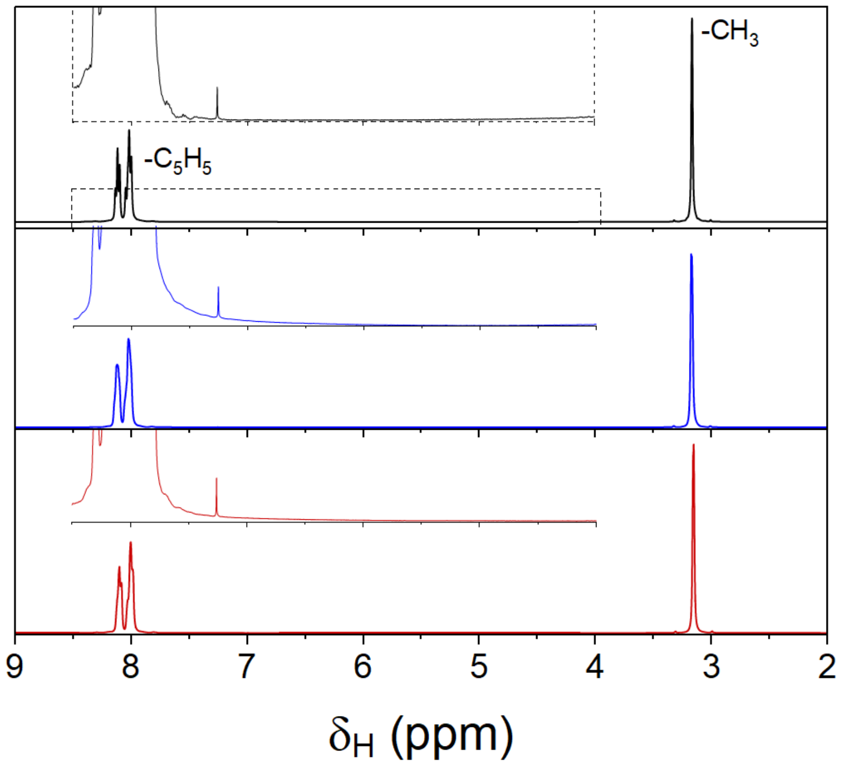

Supplementary Figure S14. ^{1}H nuclear magnetic resonance (NMR) spectra of Toluene reference sample (Black), solution of 1.5 mM $SnBr_4$ in toluene following 3 weeks exposure to ambient conditions (Blue) and 40 mM $SnBr_4$ of toluene left overnight in ambient conditions and under illumination (40 W) bulb (red). All samples were left for an additional 5 minutes under 1 sun illumination (100 mW cm^{-2}). An additional 100 µL of $CDCl_3$ was added to each solution to enable locking of the NMR and a reference signal δ_{CDCl3} = 7.26 ppm.

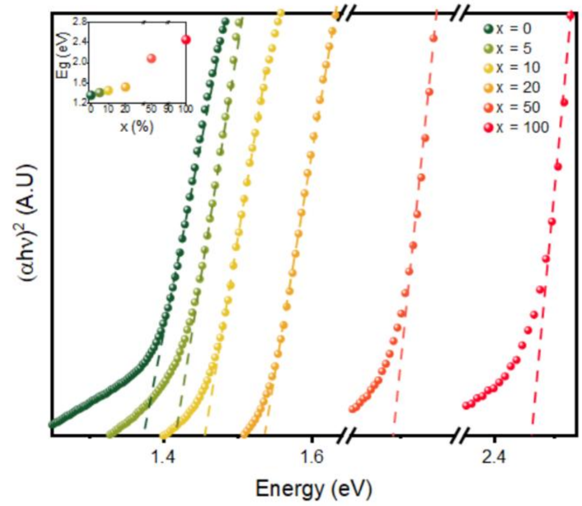

Supplementary Figure S15: Tauc plot of $PEA_{0.2}FA_{0.8}Sn(I_{100-x}Br_x)_3$ films to produce derived band gaps in **Fig. 5C**.

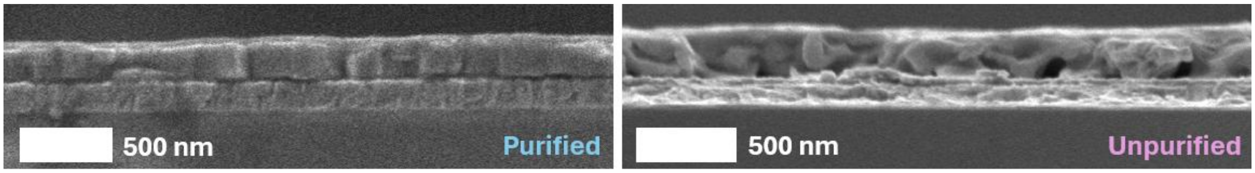

Supplementary Figure S16: Cross sectional SEM of purified and unpurified films on glass/ITO.
